# Cyclovirobuxine D Attenuates Doxorubicin-Induced Cardiomyopathy by Suppression of Oxidative Damage and Mitochondrial Biogenesis Impairment

**DOI:** 10.1155/2015/151972

**Published:** 2015-05-14

**Authors:** Qian Guo, Jiabin Guo, Rong Yang, Hui Peng, Jun Zhao, Li Li, Shuangqing Peng

**Affiliations:** ^1^Evaluation and Research Centre for Toxicology, Institute of Disease Control and Prevention, Academy of Military Medical Sciences, 20 Dongdajie Street, Fengtai District, Beijing 100071, China; ^2^Department of Biochemistry and Molecular Biology, Chongqing Medical University, Chongqing 400016, China

## Abstract

The clinical application of doxorubicin (DOX) is compromised by its cardiac toxic effect. Cyclovirobuxine D (CVB-D) is a steroid alkaloid extracted from a traditional Chinese medicine,* Buxus microphylla*. Our results showed that CVB-D pretreatment markedly attenuated DOX-induced cardiac contractile dysfunction and histological alterations. By using TUNEL assay and western blot analysis, we found that CVB-D pretreatment reduced DOX-induced apoptosis of myocardial cells and mitochondrial cytochrome c release to cytosol. CVB-D pretreatment ameliorated DOX-induced cardiac oxidative damage including lipid peroxidation and protein carbonylation and a decrease in the ratio of reduced glutathione (GSH) to oxidized glutathione (GSSG). Moreover, CVB-D was found to prevent DOX-induced mitochondrial biogenesis impairment as evidenced by preservation of peroxisome proliferator-activated receptor *γ* coactivator-1*α* (PGC-1*α*) and nuclear respiratory factor 1 (NRF1), as well as mitochondrial DNA copy number. These findings demonstrate that CVB-D protects against DOX-induced cardiomyopathy, at least in part, by suppression of oxidative damage and mitochondrial biogenesis impairment.

## 1. Introduction

Doxorubicin (DOX) is an anthracycline antibiotic which is frequently used to treat a variety of human hematological and solid tumors, such as leukemia and breast cancer [1, 2]. However, the clinical use of this potent anticancer drug is greatly limited by its concurrent adverse cardiac effect that may ultimately lead to cardiomyopathy and heart failure. It has been reported that more than a quarter of patients receiving DOX develop significant cardiac morbidity [3, 4]. The clinical symptom of DOX-induced cardiomyopathy may manifest as transient arrhythmias, nonspecific electrocardiographic abnormalities, pericarditis, and acute heart failure [5, 6]. Over the past decades, the development of pharmacological therapy for prevention and/or treatment of the cardiac toxic effect by DOX has become a critical issue in chemotherapy and cardiology. DOX cardiotoxicity involves complex multifactorial processes [5, 7]. Although the exact pathogenesis of DOX-induced cardiotoxicity is not fully understood, oxidative damage and mitochondrial biogenesis impairment have been increasingly proposed as primary mechanisms responsible for DOX-induced cardiotoxicity [2, 5]. DOX induces generation of reactive oxygen species via redox cycling during its intracellular metabolism and leads to wide spread cellular dysfunction and, ultimately, cell death in cardiomyocytes [8]. The onset and severity of DOX-induced cardiac injury have been shown to correlate with the oxidative damage and disruption of mitochondrial biogenesis [9, 10]. Studies using antioxidant therapy or transgenic mouse overexpressing antioxidant enzymes have shown protection from DOX-induced oxidative damage and cardiotoxicity, which further support a critical role of oxidative stress in DOX cardiotoxicity [11–13].

Cyclovirobuxine D (CVB-D; molecular formula: C26-H46-N2-O; molecular weight: 402.662; chemical name: 9, 19-cyclopregnan-16-ol, 4, 4, 14-trimethyl-3, 20-bis (methylamino)-, (3*β*, 5*α*, 16*α*, 20S)-) is a triterpenoid alkaloid extracted from a traditional Chinese medicine,* Buxus microphylla*, which has been widely used for hundreds of years in China to prevent and/or treat many cardiovascular diseases such as arrhythmias, heart failure, and myocardial ischemia [14–17]. As a main active ingredient of* Buxus microphylla*, increasing clinical and laboratory evidences indicate that CVB-D is an efficient protective agent in the cardiovascular system [14, 17–20]. For instance, double blind clinical trials have reported that CVB-D improves left ventricular function in coronary heart disease [20, 21]. In laboratory studies, the cardioprotective effect has been shown in animal models including dogs, pigs, and rats [14, 18, 19]. In addition, the beneficial effect of CVB-D has also been shown* in vitro* cultured ventricular cardiomyocytes and endothelial cells [14, 22, 23]. Although the mechanism of CVB-D's cardioprotective action remains poorly understood, it is increasingly reported that CVB-D may exert antioxidant property [18, 23, 24]. An* in vitro* study by Hu et al. has shown that CVB-D alleviates DOX-induced decrease of cell viability in neonatal rat cardiomyocytes [23], suggesting a protective effect of CVB-D against the cardiac injury by DOX. However, whether CVB-D is protective against DOX-induced cardiotoxicity* in vivo* is not yet clear. To this end, the present study was designed to test the ability of CVB-D on DOX-induced cardiotoxicity and further explore the mechanism by which CVB-D exerts cardioprotective effect.

## 2. Materials and Methods

### 2.1. Animals and Drug Treatments

Adult C57BL mice (18~22 g) were purchased from the Animal Center, the Academy of Military Medical Sciences (Beijing, China). Animals were housed in a ventilated animal room maintained at 23 ± 2.5°C with a standard 12/12 light/dark cycle. Food and tap water were provided* ad libitum*. All experiments were performed according to protocols approved by the Institutional Animal Care and Use Committee in compliance with the Guide for the Care and Use of Laboratory Animals published by the US National Institutes of Health. For each independent experiment, 24 mice were randomly divided into 4 groups, that is, vehicle group, CVB-D group, DOX group, and CVB-D + DOX group. Three males and three females were included in each group.

Previous studies have shown that CVB-D exerts cardioprotective effects at doses of 0.5–2.0 mg/kg/d in rodent models [25]. In clinical studies, the recommended dose of CVB-D for cardiovascular diseases is 1–6 mg/d [26]. Based on these studies, we pretreated mice daily with CVB-D (purity > 99%; ZeLang Medical technology Co., Nanjing, China; dissolved in normal saline) by gavage at a dose of 1 mg/kg/d for consecutive 4 days (cumulative dose of 4 mg/kg) or equal volume of normal saline. On the fifth day, mice were injected intraperitoneally with doxorubicin hydrochloride (HaiSun Pharmaceutical Co., Ltd., Zhejiang, China) at a dose of 15 mg/kg or equal volume of saline. Four days after DOX treatment, heart function was recorded, and mice were then euthanized by CO_2_ inhalation. Cardiac tissues were rapidly collected for further examinations.

### 2.2. Heart Function

Mouse cardiac function was evaluated by a noninvasive echocardiography. Briefly, animals were placed on a warming pad at 37°C and anesthetized by 1-2% isoflurane. Echocardiography measurements were performed by a blinded investigator using M-mode recording at mid-papillary muscle level. Two-dimensional short-axis images were obtained with a high resolution Micro-Ultrasound system (Vevo 770, VisualSonics Inc., Canada) equipped with RMV707 mechanical scan probe of 45 MHz. The left ventricular end-diastolic diameter (LVEDD) and end-systolic diameter (LVESD), fractional shortening, and ejection fraction were measured and calculated with Vevo Analysis software (version 3.0.0).

### 2.3. Histological Observation

Hearts subjected to electron microscopic examination were fixed* in situ* by vascular perfusion with normal saline for 15 min, followed by a 4% buffered formalin for 10 min. Cardiac tissues were fixed in 4% buffered formalin for at least 48 h. Afterward, cardiac tissues were embedded in paraffin and sectioned at 4 *μ*m. The sections were processed using standard histological techniques and further stained with hematoxylin-eosin. Images were captured by a digital imaging system (DP71, OLYMPUS, Japan) connected to a computerized microscopy (BX61, OLYMPUS, Japan).

### 2.4. Apoptosis Evaluation by TUNEL Assay

Paraffin-embedded sections of heart tissues were prepared and processed for a terminal deoxynucleotidyl transferase-mediated dUTP nick end labeling (TUNEL) assay using an* In Situ* Cell Apoptosis Detection Kit (Roche, Shanghai, China) according to the manufacturer's instruction. Both negative control and positive control were prepared to display accuracy and objectivity. For each sample, at least 5 fields were randomly selected from 3 slides to quantitatively analyze the apoptotic cells. Data were expressed as the percentage of apoptotic cells by calculating the ratio of positive-staining nuclei to the total number of nuclei.

### 2.5. Mitochondrion and Cytosol Isolation

Cardiac mitochondrial and cytosolic fractions were isolated from fresh collected heart by differential centrifugation as previously described [27]. In brief, heart tissues were minced and homogenized in an ice-cold extraction buffer (0.01 M Tris-HCl, 1 M mannitol, 200 mM sucrose, and 1 mM EGTA; pH 7.4). The homogenate was centrifuged twice at 600 g for 5 min to separate out the nuclear pellet. The supernatant was then centrifuged at 3,000 g for 10 min to obtain mitochondrial pellet, and the resulting supernatant was centrifuged at 14,000 g for 10 min to obtain cytosolic pellet. These pellets were resuspended and spun again to purify mitochondrial and cytosolic fractions. These isolated fractions were further used for western blot analysis of cytochrome c. The purity of isolated fraction was confirmed by western blot analysis of porin and histone H1 markers, respectively, for mitochondria and nuclei, to show the absence of mitochondrial and nuclear contamination in cytosol fraction and nuclear contamination in mitochondrial fraction.

### 2.6. Lipid Peroxidation

Cardiac lipid peroxidation was quantified by measuring the formation of thiobarbituric acid reactive substances (TBARS) as we previously described [28]. The TBARS content was measured spectrophotometrically at 532 nm and calculated based on a standard curve using 1,1,3,3-tetraethoxypropane as a standard. Data were expressed as nanomoles per milligram of protein. Protein concentration in the samples was measured by a bicinchoninic acid commercial kit (Biyuntian Co., Hangzhou, China), using bovine serum albumin as a standard.

### 2.7. Protein Carbonylation

Protein carbonyl content in cardiac tissues was determined according to Floor and Wetzel with slight modification [29]. Briefly, homogenate prepared from fresh heart was reacted with 10 mM 2,4-dinitrophenylhydrazine in 2 M HCl for 1 h at room temperature, precipitated with 6% trichloroacetic acid, and suspended in ethanol/ethyl acetate (1 : 1). Proteins were then solubilised in 6 M guanidine hydrochloride and 50% formic acid was added and centrifuged at 14,000 g for 10 min to remove any trace of insoluble material. Carbonyls were measured spectrophotometrically at 370 nm. Data were expressed as nmol of 2,4-dinitrophenyl-hydrazine incorporated/mg protein based on the molar extinction coefficient of 22,000 M^−1^ cm^−1^.

### 2.8. GSH/GSSH Ratio Determination

Total soluble reduced glutathione (GSH) and oxidized glutathione (GSSG) were measured in fresh heart homogenate using a commercial kit (Jiancheng Bio Ins., Nanjing, China) according to the manufacturer's instruction. Briefly, cardiac tissue homogenates were added to GSH or GSSH assay buffers. For GSH sample, a reaction mixture was added containing NADPH, 5,5′-dithio-2-nitrobenzoic acid (DTNB), and glutathione reductase. The rate of DTNB reduction was monitored at the absorbance of 412 nm. For detection of GSSG content, the thio-scavenging reagent 1-methy-2-vinylpyridinium trifluoromethanesulfonate was immediately mixed with the samples to eliminate GSH. GSH and GSSG concentrations were calculated by linear regression against the standard curve and normalized to protein content, and GSH/GSSG ratio was calculated as [(GSH − 2GSSG)/GSSG].

### 2.9. Western Blot Analysis

Protein samples were extracted from cardiac tissues. Afterward, extracted proteins were electrophoresed and separated on a 12% SDS-polyacrylamide gel and transferred to a polyvinylidene fluoride membrane (Millipore, Billerica, USA). The membrane was incubated overnight with specific primary antibodies against cytochrome c (Santa Cruz; dilution, 1 : 2000); PGC-1*α* (Abcam, UK; dilution, 1 : 1000); and nuclear respiratory factor 1 (NRF-1) (Abcam; dilution, 1 : 1000), TFAM (Abcam; dilution, 1 : 1000), MnSOD (Abcam; dilution, 1 : 1000), UCP2 (Abcam; dilution, 1 : 1000), and *β*-actin (Santa Cruz; dilution, 1 : 5000). After being washed in Tris-buffered saline (pH 7.2) containing 0.05% Tween 20, the membranes were incubated with the secondary antibody for 2 h at room temperature. Blots were developed with electrochemiluminescence, and proteins were quantified by densitometry using *β*-actin as a loading control. Data from at least three independent experiments were collected for quantitative analysis.

### 2.10. mtDNA Copy Number

Total DNA was isolated from heart tissues and the relative mtDNA copy number was determined by qPCR as previously described [30]. The cytochrome c-oxidase subunit I gene of the mitochondrial DNA (mtDNA) was quantified and normalized against NDUFV1 nDNA gene. The cytochrome c-oxidase subunit I primers were 5-TGCTAGCCGCAGGCATTAC-3 (forward primer) and 5-GGGTGCCCAAAGAATCAGAAC-3 (reverse primer). The NDUFV1 primers were 5-CTTCCCCACTGGCCTCAAG-3 (forward primer) and 5-CCAAAACCCAGTGATCCAGC-3 (reverse primer). qPCR was performed on multiplex real-time fluorescence quantitative PCR (iQ 5, BIORAD, USA) using PCR Supermix (Invitrogen). The relative mtDNA to nDNA ratio in each sample was determined based on amplification curves.

### 2.11. Statistical Analysis

Statistical analysis was performed using GraphPad Prism 5.0. Data were expressed as mean ± standard error of at least three independent experiments. Comparison between groups was determined by one-way analysis of variance followed by paired *t*-test or 2 × 2 factorial design analysis. A level of *P* < 0.05 was considered statistically significant.

## 3. Results

### 3.1. CVB-D Reduces DOX-Induced Cardiac Contractile Dysfunction and Histological Alterations

**Figure 1:**
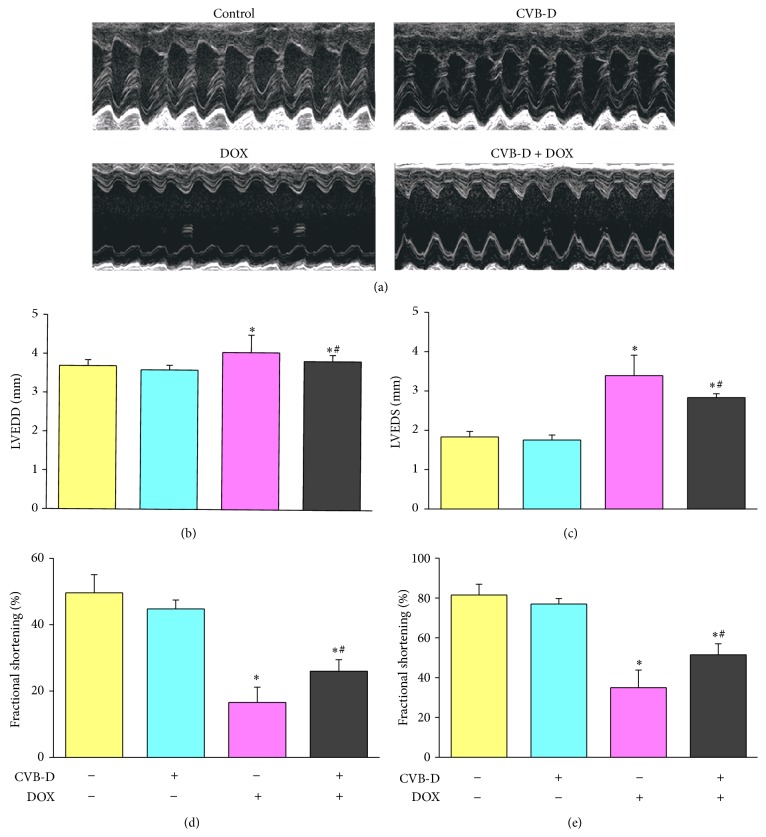
CVB-D attenuates DOX-induced cardiac contractile dysfunction. (a) Representative mouse short-axis echocardiograms. Cardiac function parameters were indicated by (b) LVEDD and (c) LVESD, (d) ejection fraction, and (e) fractional shortening. ^∗^ *P* < 0.05 versus the control group; ^#^ *P* < 0.05 versus the DOX group, *n* = 6.

Numerous studies have shown that DOX may induce heart contractile dysfunction soon after administration [2, 31]. In the present study, mouse heart function was monitored by echocardiography four days after a single administration of DOX. In our preliminary experiments, we have compared the echocardiographic data for each animal before/after CVB-D treatment. No significant difference was found between CVB-D group and vehicle group, and no obvious change was found after CVB-D administration compared to predose data (data not shown). Representative two-dimensional M-mode tracings of left ventricular wall motion were shown in Figure 1(a). Compared with the control, the range of waveforms was significantly decreased by DOX, and this change was reduced by pretreatment with CVB-D. By quantitative analysis of these echocardiograms, we found that CVB-D alone has no effect on heart function in physiological condition. DOX induced significant increases of 30.7% and 85.4%, respectively, for LVEDD and LVEDS and decreases of 57.1% and 65.0%, respectively, for ejection fraction and fractional shortening. Compared to DOX treated mice, mice treated with CVB-D plus DOX showed improved heart function as indicated by lower LVEDD and LVEDS and higher ejection fraction and fractional shortening.

**Figure 2:**
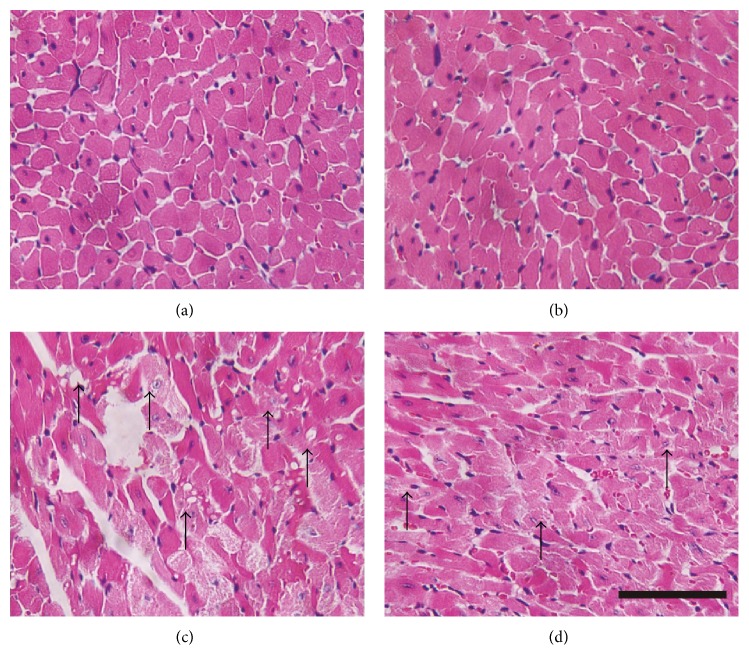
Light micrograph demonstrating the effect of CVB-D on DOX-induced myocardial histological alterations. Representative photomicrographs of mouse heart stained with H&E. (a) Normal saline-treated control; (b) CVB-D pretreated group; (c) DOX-treated group; (d) CVB-D plus DOX-treated group. Arrows indicate areas of histological changes including reduced myofibrils, swelling, vacuolization, and nuclear condensation or dissolution. Scale bar = 20 *μ*m.

To demonstrate cardiac morphological alterations, sections of mouse heart tissue stained with hematoxylin-eosin were examined by light microscopy. As shown in Figure 2, heart section from control showed normal cardiac morphology. DOX induced obvious myocardial pathology including reduced myofibrils, swelling, vacuolization, and nuclear condensation or dissolution. CVB-D by itself had no effect on cardiac morphology; however, pretreatment with CVB-D significantly ameliorated DOX-induced lesions on myocardial morphology. DOX-induced cardiotoxicity has been shown to manifest similar pathophysiological characteristics with myocardial infarction-induced cardiac injury, such as heart dysfunction and morphology alterations. Thus, these findings are consistent with the recent studies which indicate that CVB-D provides efficient protection against myocardial infarction-induced cardiac dysfunction and pathological changes.

### 3.2. CVB-D Inhibits DOX-Induced Myocardial Apoptosis and Mitochondrial Cytochrome c Release

**Figure 3:**
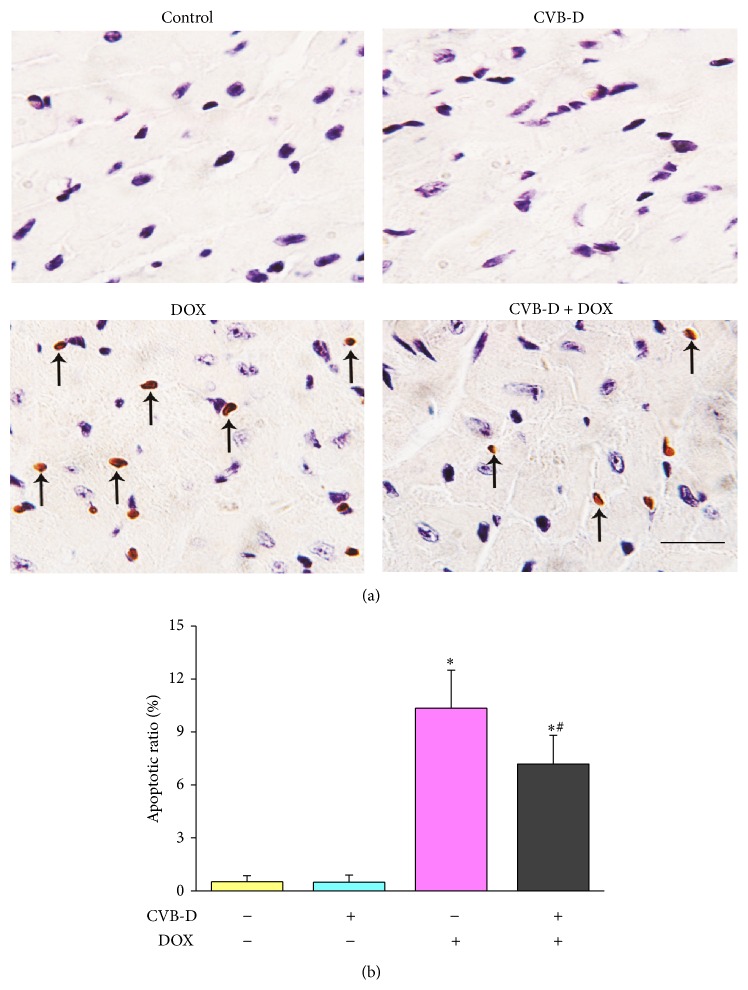
CVB-D relieves DOX-induced apoptosis in cardiac cells. Representative photomicrographs of TUNEL stained heart section. (a) Arrows indicate TUNEL-positive cells, scale bar = 20 *μ*m. (b) Quantitative analysis of the percentage of apoptotic cells.

Apoptosis of cardiac cells has been suggested to be critically involved in the development of myocardial loss and severe contractile dysfunction during the pathogenesis of DOX cardiomyopathy [10, 32]. By application of TUNEL assay, a significant increase of apoptotic cells was found in heart section from DOX group. Mice in CVB-D group and vehicle group showed similar apoptotic ratio which is under 1%, indicating that CVB-D had no effect on myocardial cell apoptosis* in vivo*. In contrast, CVB-D pretreatment effectively alleviated DOX-induced cardiac apoptosis. The apoptotic ratio was about 10.35% and 7.1%, respectively, for DOX and CVB-D + DOX group (Figure 3).

**Figure 4:**
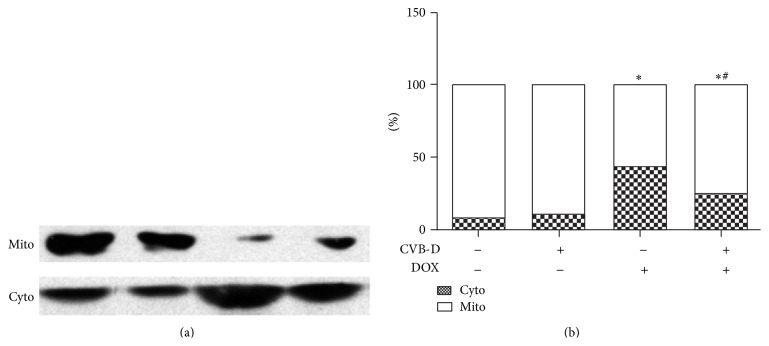
CVB-D inhibits cytochrome c release from mitochondria into cytosol in the heart. (a) Representative blots of cytochrome c in cardiac mitochondrial and cytosolic fractions. (b) Quantitative analysis of cytochrome c protein expression. ^∗^ *P* < 0.05 versus the control group; ^#^ *P* < 0.05 versus the DOX group.

Mitochondrial cytochrome c release into cytosol plays a pivotal role in DOX-induced apoptosis. As shown in Figure 4, western blot analysis revealed that DOX treatment significantly increased cytosolic concentrations of cytochrome c with a decrease in mitochondria. Pretreatment with CVB-D preserved cytochrome c in mitochondria against DOX, as evidenced by significantly improved cytochrome c release into cytosol in CVB-D + DOX group. CVB-D alone had no effect on the distribution of cardiac cytochrome c in mitochondria and cytosol. These findings are consistent with the results from TUNEL assay and correlate with cardiac function alterations.

### 3.3. CVB-D Ameliorates DOX-Induced Oxidative Damage

**Figure 5:**
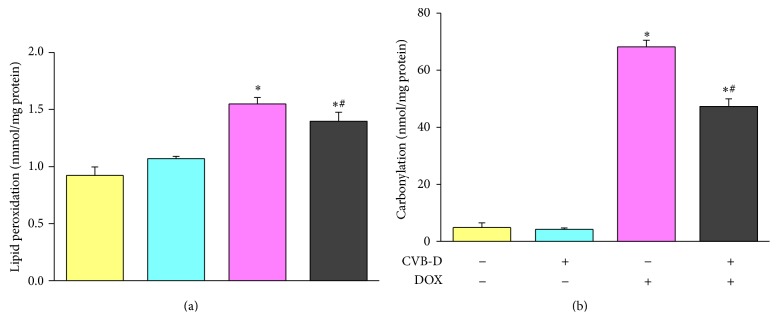
CVB-D protects against DOX-induced cardiac oxidative damage indicated by (a) lipid peroxidation and (b) protein carbonylation. ^∗^ *P* < 0.05 versus the control group; ^#^ *P* < 0.05 versus the DOX group, *n* = 6.

DOX-induced oxidative damage was evaluated by determination of lipid peroxidation and protein carbonylation. As shown in Figure 5, we did not observe significant difference in the level of TBARS in normal saline-treated mice and CVB-D treated mice. DOX treatment significantly increased cardiac TBARS level, and this effect was ameliorated by CVB-D pretreatment. As compared to that in normal saline-treated mice, the cardiac TBARS level in DOX-treated mice and CVB-D + DOX-treated animals was increased by 68.0% and 51.4%, respectively (Figure 4(a)). Similar results were found in the assessment of cardiac protein oxidative injury. CVB-D by itself had no effect on protein carbonylation but significantly inhibited DOX-induced protein carbonyl accumulation. The cardiac protein carbonyl contents were found to be increased by 13.0- and 10.2-fold, respectively, for DOX-treated mice and CVB-D + DOX-treated animals compared to that in controlled mice (Figure 4(b)).

**Figure 6:**
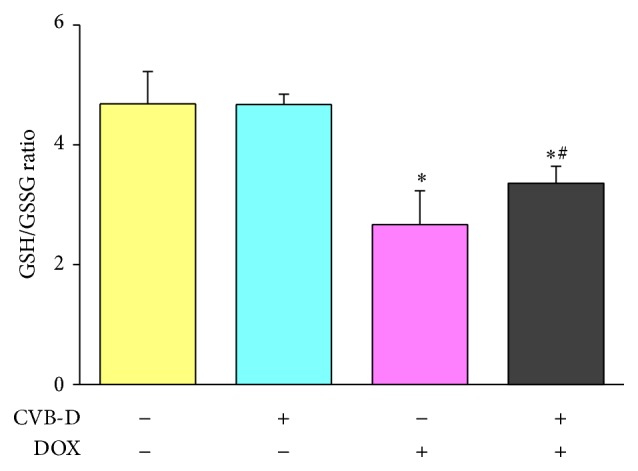
CVB-D alleviates DOX-induced decrease of GSH/GSSG ratio. ^∗^ *P* < 0.05 versus the control group; ^#^ *P* < 0.05 versus the DOX group, *n* = 6.

The antioxidant defense system is an important factor determining the fate of DOX-induced oxidative injury [5, 31, 33]. As one of the most important antioxidants in the heart, GSH plays a key role in the detoxification of a variety of chemicals including DOX [34]. Maintaining an optimal GSH/GSSG ratio is critical to the survival of cardiac cells and proper heart function. The ratio of GSH to GSSG has been widely used as a marker for oxidative stress in the cardiovascular system [35, 36]. In the present study, GSH/GSSG ratio was decreased by 43.0% in DOX-treated mice as compared to normal saline-treated mice. CVB-D by itself did not affect the ratio of GSH/GSSG in the heart under physiological condition. Pretreatment with CVB-D significantly prevented DOX-induced reduction of GSH/GSSG ratio. The cardiac GSH/GSSG ratio in CVB-D + DOX-treated mice was 1.26-fold higher than that in DOX-treated mice (Figure 6).

### 3.4. CVB-D Improves Cardiac Mitochondrial Biogenesis Impairments by DOX

**Figure 7:**
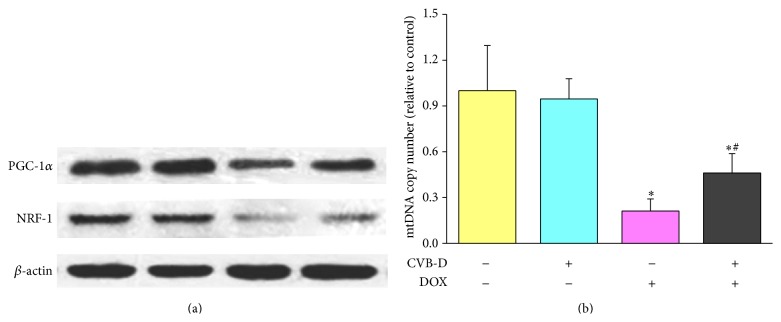
CVB-D ameliorates DOX-induced mitochondrial biogenesis impairment. (a) Representative blots of cardiac PGC-1*α* and NRF-1; (b) mtDNA copy number determined by qPCR. ^∗^ *P* < 0.05 versus the control group; ^#^ *P* < 0.05 versus the DOX group.

A growing body of evidence suggests that impaired cardiac mitochondrial biogenesis plays a key role in DOX cardiotoxicity [37–39]. PGC-1*α* is emerged as a master regulator for mitochondrial biogenesis. PGC-1*α* interacts with NRFs, to activate mitochondrial biogenesis by facilitating transcription, translation, and activation of downstream transcription factors necessary for mtDNA replication [40]. In the present study, the expression of PGC-1*α* and NRF-1 as well as mtDNA copy number was determined to evaluate the effect of CVB-D on DOX-induced cardiac mitochondrial biogenesis impairment. As shown in Figure 7(a), CVB-D pretreatment has no obvious effect on the protein expression of cardiac PGC-1*α* and NRF-1. However, CVB-D was found to effectively improve DOX-induced decreased expression of both PGC-1*α* and NRF-1. These changes were well mirrored by the results from the assay of mtDNA copy number, one of the most frequently used markers for mitochondrial biogenesis. As compared to the mice in control group, cardiac mtDNA copy number of DOX-treated mice was decreased by 78.8%, while mice treated with CVB-D plus DOX showed a significant ameliorated mtDNA injury with a reduction of 54.0% (Figure 7(b)).

## 4. Discussion

DOX has been used in chemotherapy as one of the most effective anticancer agents since the late 1960s. Despite its efficacy, the application of DOX is greatly compromised by its cardiotoxicity. Significant efforts have been made in the past decades aiming at prevention and/or attenuation of DOX-induced cardiotoxicity, but, so far, the efficiency of these interventions is limited [1, 5, 41]. The present study showed for the first time that CVB-D pretreatment significantly improved DOX-induced cardiac contractile dysfunction, histological alterations, apoptosis, and mitochondrial cytochrome c release to cytosol. Pretreatment with CVB-D ameliorated DOX-induced cardiac oxidative damage including lipid peroxidation and protein carbonylation and a decrease in GSH to GSSG ratio. Moreover, CVB-D was found to prevent DOX-induced mitochondrial biogenesis impairment as indicated by preservation of PGC-1*α* and NRF-1, as well as mitochondrial DNA copy number. These findings demonstrate that CVB-D protects against DOX-induced cardiomyopathy, at least in part, by suppression of oxidative damage and mitochondrial biogenesis impairment.

CVB-D has been increasingly shown to provide efficient protection against many cardiovascular diseases including myocardial infarction-induced heart failure [14, 18, 19]. By using primary cultured neonatal rat cardiomyocytes, Hu and colleagues previously showed that CVB-D pretreatment can reduce DOX-induced decrease of cell viability [23]. However, because of the much more complicated environment in* in vivo* where systemically metabolic, hormonal, and neuronal influences as well as compensatory effects cannot be excluded, it is necessary to testify whether CVB-D is effective in protecting against DOX cardiotoxicity* in vivo*. Our results showed that CVB-D by itself has no obvious effects on cardiac function which is consistent with previous finding [42]; however, mice pretreated with CVB-D were well protected from DOX-induced cardiomyopathy as evidenced by significant improved cardiac contractile dysfunction, histological alterations, and apoptosis. DOX-induced cardiomyopathy is a complex multifactorial process that leads to heart damage and dysfunction, which is mediated by a series of events involving production of reactive oxygen species, disruption of mitochondrial biogenesis, energy depletion, alteration of ionic homeostasis, and cell death by apoptosis. These characteristics of DOX-induced are highly similar to that observed in ischemic heart failure [1, 7]. Thus, our findings are in agreement with the previous studies showing that CVB-D protects the heart from myocardial infarction-induced injuries [18, 23]. Though no obvious changes by CVB-D was found under physiological conditions, CVB-D may somewhat induce effects that are beneficial for the cardiovascular system to be protected from damage. Another possible explanation is that CVB-D may play some role in maintaining the homeostasis and preservation of cardiac function and exerts protective effects under pathological condition.

Cardiac cells contain abundant mitochondria which have been implicated to play a central role in the pathogenesis of cardiac injury [43]. DOX has a high affinity for cardiolipin which localizes in the inner membrane of mitochondria, leading to specific accumulation of DOX in cardiac mitochondria [7, 44]. Increasing evidence suggests that DOX cardiotoxicity is mainly mediated by reactive oxygen species generation within mitochondria. During the metabolism, DOX undergoes one-electron reduction that is catalyzed by NAD(P)H reductases to yield a semiquinone free-radical intermediate, which regenerates its parent quinone by reacting with O_2_ to produce superoxide anion and other forms of reactive oxygen species [8]. As a main site for bioreductive metabolism of DOX and reactive oxygen species generation, mitochondria are also preferential targets of reactive oxygen species. Excess reactive oxygen species can attack the components of mitochondria, to induce mitochondrial dysfunction leading to oxidative damage, and ultimately initiate cell death such as apoptosis [44, 45]. Our results showed that CVB-D pretreatment significantly improved DOX-induced cardiac oxidative damage as evidenced by ameliorated lipid peroxidation and protein carbonylation. These results are in agreement with previous report showing a lipid peroxidation inhibitory activity of CVB-D and its derivatives [24]. In addition, our results showed that CVB-D protected GSH, a predominant antioxidant in detoxification of DOX-induced oxidative stress. Because reactive oxygen species are very active and are easily metabolized, it is particularly not easy to precisely detect the level of reactive oxygen species in* in vivo* studies. Although our findings are not direct evidence demonstrating the effect of CVB-D on reactive oxygen species generation, they strongly suggest that CVB-D protect the heart from DOX toxicity by suppressing oxidative damage and preserving antioxidant capacity.

Mitochondria in cardiac cells are highly dynamic involving mitochondrial biogenesis which is crucial for energetic metabolism and maintaining a proper function of heart [40, 46, 47]. In particular, mitochondrial biogenesis is important for overcoming mitochondrial damage and repairing cell injury under pathological conditions [40, 46]. DOX has been shown to inhibit PGC-1*α*, a master regulator of mitochondrial biogenesis, and affect the transcription of mitochondrial genome, consequently leading to disruption of mitochondrial biogenesis [10, 37]. Targeting at preservation of PGC-1*α* and mitochondrial biogenesis by many pharmacological agents, including traditional Chinese medicines like QishenYiQi pills, has been shown to provide efficient protection against DOX-induced cardiac injuries [31, 48, 49]. Our results showed that, under physiological conditions, CVB-D neither affects the protein expression of PGC-1*α* nor alters the cardiac mtDNA content. However, CVB-D pretreatment remarkably inhibited DOX-induced depression of PGC-1*α* and NRF-1 protein expression. Consistent with these findings, CVB-D was also found to effectively prevent the decrease of mtDNA number by DOX, suggesting that the cardioprotective effect of CVB-D is associated with preservation of mitochondrial biogenesis.

In conclusion, the present study depicts a novel function of CVB-D in protection against DOX-induced cardiotoxicity and suggests that the cardioprotective action of CVB-D is mediated, at least in part, by suppression of oxidative damage and mitochondrial biogenesis impairment. Given the facts that CVB-D has been proven as an efficient protective agent against cardiovascular diseases like heart failure and lack of preventive or therapeutic options for the toxic cardiomyopathy of DOX, our data indicate that CVB-D may clinically provide a promising cardioprotection against DOX cardiotoxicity. However, many critical questions need further investigations. For example, pharmacokinetics of CVB-D in the current study is unrevealed. It is not clear whether CVB-D affects the antitumor activity of DOX. Future studies aiming at these questions will be important and informative for the clinical application of CVB-D in protecting patient from DOX-induced cardiac side effects during chemotherapy.

## Abbreviations

CVB-D: Cyclovirobuxine D
DOX: Doxorubicin
DTNB: 5,5′-Dithio-2-nitrobenzoic acid
GSH: Reduced glutathione
GSSG: Oxidized glutathione
LVEDD: Left ventricular end-diastolic diameter
LVEDS: Left ventricular end-systolic diameter
mtDNA: Mitochondrial DNA
NRF-1: Nuclear respiratory factor 1
PGC-1*α*: Peroxisome proliferators–activated receptor *γ* coactivator-1 *α*
TBARS: Thiobarbituric acid reactive substances
TUNEL: Terminal deoxynucleotidyl transferase-mediated dUTP nick end labeling.
